# Maturation of the Language Network: From Inter- to Intrahemispheric Connectivities

**DOI:** 10.1371/journal.pone.0020726

**Published:** 2011-06-13

**Authors:** Angela D. Friederici, Jens Brauer, Gabriele Lohmann

**Affiliations:** 1 Department of Neuropsychology, Max Planck Institute for Human Cognitive and Brain Sciences, Leipzig, Germany; 2 Department of Neurophysics, Max Planck Institute for Human Cognitive and Brain Sciences, Leipzig, Germany; University of Barcelona, Spain

## Abstract

Language development must go hand-in-hand with brain maturation. Little is known about how the brain develops to serve language processing, in particular, the processing of complex syntax, a capacity unique to humans. Behavioral reports indicate that the ability to process complex syntax is not yet adult-like by the age of seven years. Here, we apply a novel method to demonstrate that the basic neural basis of language, as revealed by low frequency fluctuation stemming from functional MRI data, differs between six-year-old children and adults in crucial aspects. Although the classical language regions are actively in place by the age of six, the functional connectivity between these regions clearly is not. In contrast to adults who show strong connectivities between frontal and temporal language regions within the left hemisphere, children's default language network is characterized by a strong functional interhemispheric connectivity, mainly between the superior temporal regions. These data indicate a functional reorganization of the neural network underlying language development towards a system that allows a close interplay between frontal and temporal regions within the left hemisphere.

## Introduction

The neural tissue supporting language processing in the adult brain has long been located in the left inferior frontal cortex [1] and the temporal cortex [2]. The particular functions of these regions during language processing have been specified over the past decades, leading to articulated models of the functional neuroanatomy of language in the mature brain [3], [4], [5], [6]. All of these models assume that inferior frontal and temporal regions of the left hemisphere are involved during language processing, although their particular contributions are still a matter of debate.

Structurally, the interaction between inferior frontal and temporal regions must be based either on direct cortico-cortical fibers or thalamocortical reciprocal pathways. A number of recent studies using diffusion tensor imaging (DTI) methods have identified fiber tracts connecting the language-relevant areas in the inferior frontal and the temporal cortex in vivo [7], [8]. These studies report two pathways: a ventral pathway connecting the ventral part of the inferior frontal gyrus (IFG) to the anterior-to-mid portion of the superior temporal gyrus (STG) via the uncinate fasciculus and/or the extreme capsule fiber system, and a dorsal pathway connecting the dorsal part of the IFG to the posterior portion of the STG and sulcus (STG/STS) via the superior longitudinal fasciculus and the arcuate fasciculus [9], [10], [11], [12], [13]. During development the dorsal pathway matures late [14], [15], [16] and still has not fully matured by the age of seven years [17].

Studies that combined DTI with functional magnetic resonance imaging (fMRI) attributed different functions to the different pathways, in particular the dorsal one. While one study [11] found the dorsal pathway connected regions which support the processing of complex syntax, another study [13] takes this pathway as supporting language repetition by auditory-motor mapping. As the dorsal pathway described in each of the two studies differed with respect to its endpoints in the frontal cortex, it is possible that there are two parallel-running fiber bundles. It is interesting to note that within the inferior frontal cortex, there are short-range structural connectivities between the inferior frontal sulcus (IFS, located dorsally to the pars opercularis) and Broca's area in the IFG [18]. Functionally, these two regions have been attributed to working memory and syntactic hierarchization, which interact during the processing of syntactically complex sentences [18]. Moreover, analyses of long-range functional connectivities during language comprehension report strong correlations between the IFG and the posterior temporal cortex and attributed these to specific language processes investigated by different experimental conditions in the respective studies, namely syntax or semantics [13], [19], [20]. It has been argued, however, that different linguistic conditions investigated in fMRI studies only explain a very small part of the overall variance [21] and that much of the variance is buried in the low frequency fluctuations of these studies.

As an alternative approach, the functional properties of a network can be investigated by correlational methods based on the analysis of low frequency fluctuations (LFF) [21]. Such an analysis can provide insight into the fundamental functional connections within the brain, as LFF (<0.1 Hz) amplitudes represent a large portion of the overall signal variance of the BOLD response as measured in the fMRI. This type of analysis has previously been successfully applied in resting state studies [22], [23], [24], [25], [26]. A recent study used a novel approach and employed an LFF analysis to language experiments with the goal of determining the functional connectivities between inferior frontal and temporal regions, independent of the particular language condition and tasks [27]. By comparing LFF of a number of language experiments to those of non-language experiments, a particular language network was identified. This network contained a strong correlation between the ventral part of the IFG and STS, and also between the dorsal part of the IFG bordering the IFS and the posterior STS in the left hemisphere. In contrast to a recent study investigating resting state which seeded in different subregions of the IFG, and found a correlation between the IFG and parietal regions [25], no such correlation was found in the LFF analysis of the data from the language study. These findings indicate that the LFF network underlying language is different from that underlying resting-state. As the observed frontal-to-temporal correlations in the language studies were independent of the different stimuli and tasks used in four different language studies, the correlational network was taken to represent the basic language network. In analogy to the default mode network observed during resting state [22], [23], [24], [25], [26], [27], [28], we called the network underlying language studies the “default language network”.

Here, we show that the default language network in children differs considerably from that of adults. Based on structural connectivity data obtained in children and adults we expected the language network of children to differ from that of adults not only structurally, but also functionally. Structural studies indicate that the dorsally located fiber track which connects the left IFG and the left posterior superior temporal cortex only matures late during development [14], [15], [16], and has still not fully matured at seven years of age [17]. Therefore, we hypothesized that frontal-to-temporal functional connectivities within the default language network should increase during development.

We investigated this issue using LFF data from fMRI experiments on language processing in six-year-old children and adults using an established paradigm on auditory sentence comprehension [27], [29], [30]. The LFF approach allowed us to determine the default language network in the different age groups, independent of the various aspects manipulated in the experimental conditions. We choose two seeds in the inferior frontal cortex, namely the IFG and the IFS since both regions have been shown to activate during the processing of complex sentences; the IFG as a function of syntactic processes in adults [11], [18], [30], [31], [32], [33], [34], [35], [36], [37], [38], and IFS as a function of working memory resources during the processing of syntactically complex sentences [18]. Therefore, in the present study, we performed a hypothesis driven correlational analyses investigating the functional connectivity between the left inferior frontal cortex and the posterior STG/STS for two adjacent regions, namely BA 44 as part of the IFG, and the IFS. Moreover, analyses were conducted seeding in the left posterior STS.

## Results

**Figure 1:**
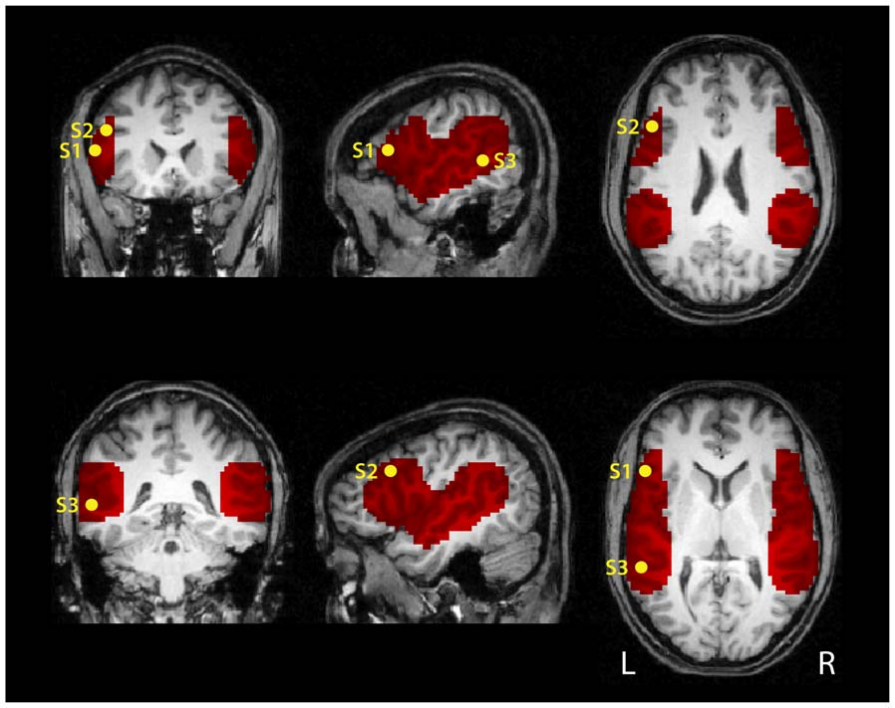
Location of seeds and their volume of interest mask used for the functional connectivity analysis. The volume of interest (VOI) was selected to cover perisylvian language areas in inferior frontal (IFG/IFS) and posterior temporal (STG/STS) cortices. Seed regions are indicated inside the mask: seed 1 (S1) at −53, 20, 15 (Talairach coordinates) in left IFG, seed 2 (S2) at −47, 20, 30 in left IFS and seed 3 (S3) at −56, −43, 9 in left STS. Each seed region covered 7 voxels (189 mm^3^). The VOI comprised a total of 6224 voxels (168,048 mm^3^). The data in all figures are overlaid onto a T1-weighted adult template in Talairach atlas space.

Functional connectivity results from three seeds were calculated for each age group: seed 1 in left BA 44 (Talairach coordinates −53, 20, 15), seed 2 in left IFS (−47, 20, 30) and seed 3 in left STS (−56, −43, 9). Since a clear hypothesis for particular brain areas was formulated, statistical examination concentrated on perisylvian language areas in the inferior frontal and superior temporal cortices within both hemispheres. These areas were selected to form a volume of interest (VOI). For seed locations and VOI, see Figure 1.

### Correlations with seed in the frontal cortex

**Figure 2:**
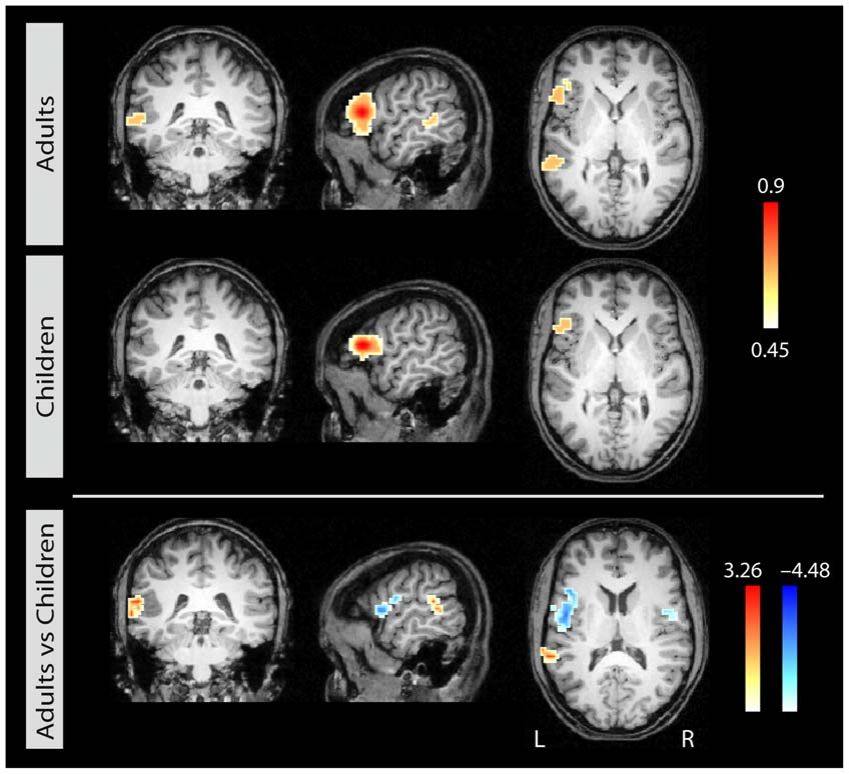
Correlational maps for each age group for seed 1 in left BA 44. Group average correlational maps (*r*-values, thresholded at 0.45) for adults (first row) and for children (second row). The third row displays the statistical difference (*z*-values) between adults (red) and children (blue). Colored regions indicate a statistically significant difference between the groups (p<0.05, corrected). Left column: coronal view; middle column: sagittal view; right column: axial view.

When seeded in left BA 44, strong correlations were obtained with the left posterior temporal cortex in adults whereas in children, no such ipsilateral correlation was observed. Figure 2 shows group averages of correlation maps generated using the seed voxel in BA 44. For all figures, the inverse of the Fisher r-to-z transform was applied to the averages so that the maps show correlation values and not their transforms. The lower row in Figure 2 displays the result of the t-test comparing the two age groups directly. This contrast reveals that instead of an ipsilateral correlation with the temporal cortex, children show stronger correlations of BA 44 with the contalateral inferior frontal region (see also Table 1).

**Table 1:** Group differences of correlation patterns for the three seed regions.

Region	Contrast	Location (x y z)	Maximum	Size
**Seed 1: left IFG**				
L pSTG/STS	Adults > Children	−57 −39 18	3.27	891
L IFG/PCG	Children > Adults	−39 15 12	−4.49	3942
R IFG/PCG	Children > Adults	39 0 12	−3.53	1026
**Seed 2: left IFS**				
L pSTG/STS	Adults > Children	−57 −45 12	4.16	4212
L IFG	Adults > Children	−54 27 3	3.45	513
L PCG	Children > Adults	−48 −6 15	−3.66	1188
R PCG	Children > Adults	51 3 36	−2.95	486
R pSTG/SMG	Children > Adults	57 −36 33	−2.90	513
**Seed 3: left STS**				
L IFG/IFS	Adults > Children	−51 15 33	4.80	1539
L IFG	Adults > Children	−54 27 3	3.45	702
L pSTS	Children > Adults	−36 −39 15	−4.78	3456
L mSTS	Children > Adults	−54 −18 −9	−3.12	432
R pSTG/STS	Children > Adults	45 −45 18	−3.51	3024

List of correlation clusters for the direct group contrast for each of the three seed regions (cf. Figure 2–4). Only clusters >300 mm^3^ are listed with region labels, contrast, location (Talairach coordinates), maximum z-value, and size in mm^3^ (p<0.05, corrected). L = left, R = right, p = posterior, m = middle, PCG = precentral gyrus, SMG = supramarginal gyrus.

**Figure 3 pone-0020726-g003:**
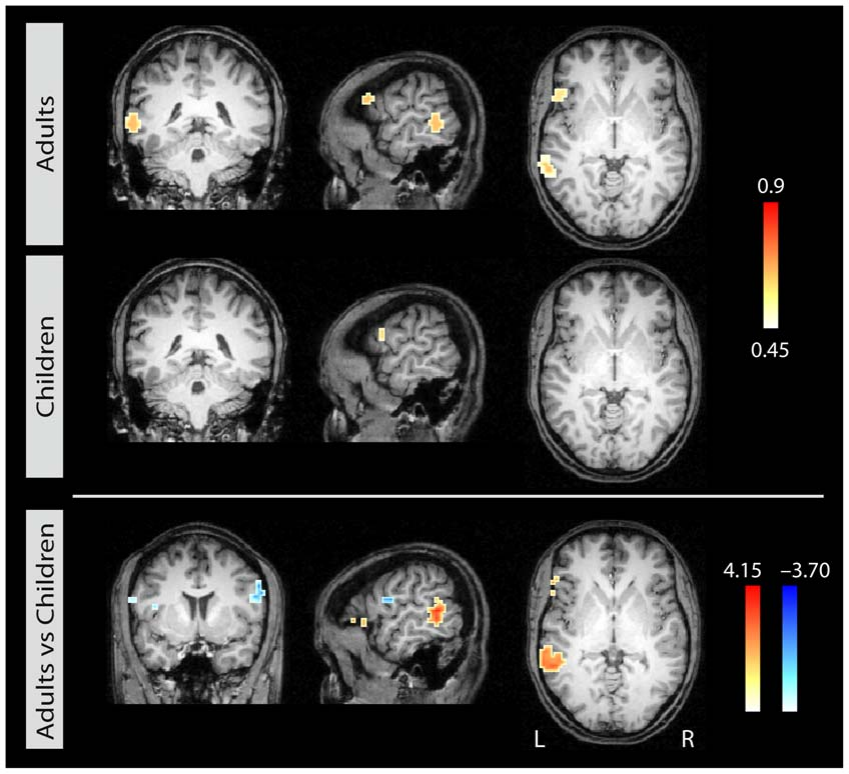
Correlational maps for each age group for seed 2 in left IFS. Group average correlational maps (*r*-values, thresholded at 0.45) for adults (first row) and for children (second row). The third row displays the statistical difference (*z*-values) between adults (red) and children (blue). Colored regions indicate a statistically significant difference between the groups (p<0.05, corrected). Left column: coronal view; middle column: sagittal view; right column: axial view.

When seeded in the left IFS, we observe a strong correlation with the posterior STG/STS in adults, but not in children (see Figure 3). The results of the t-test comparing the two age groups directly are displayed in the lower row of Figure 3. Again, the direct contrast reveals that children, in contrast to adults, show a correlation with the precentral gyri in both hemispheres and also the right posterior temporal region.

### Correlations with seed in the posterior temporal cortex

**Figure 4:**
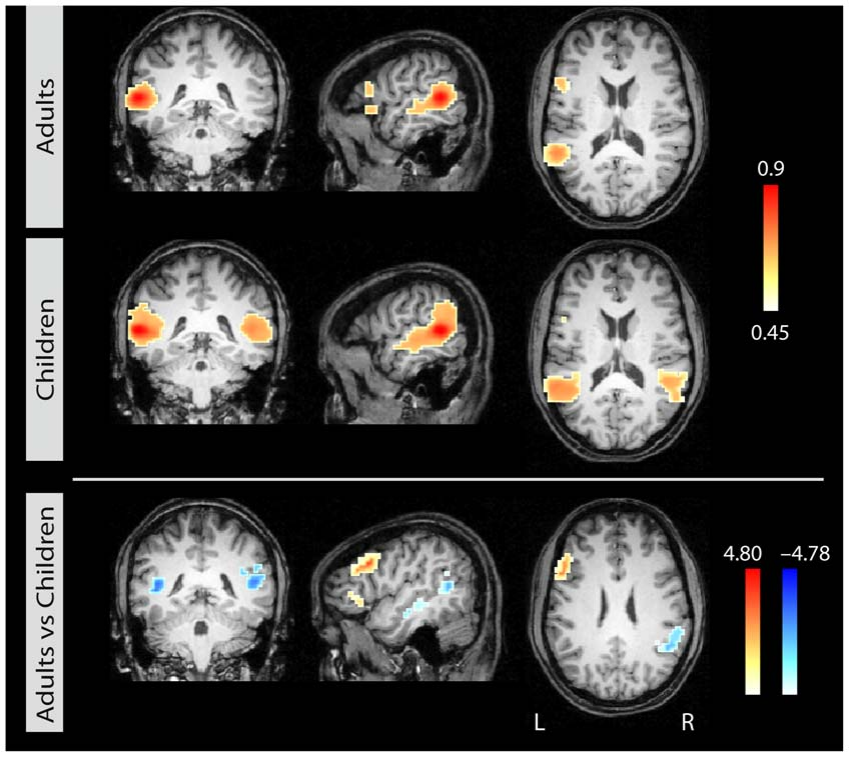
Correlational maps for each age group for seed 3 in left STS. Group average correlational maps (*r*-values, thresholded at 0.45) for adults (first row) and for children (second row). The third row displays the statistical difference (*z*-values) between adults (red) and children (blue). Colored region indicate a statistically significant difference between the groups (p<0.05, corrected). Left column: coronal view; middle column: sagittal view; right column: axial view.

When seeded in the left posterior STS, stronger correlations with left IFG and both BA 44 and the IFS were found in adults compared to children. For children, in contrast to adults, the analysis revealed strong correlations with the contralateral temporal region. This is confirmed in the direct contrast in the lower row of Figure 4 showing the result of the t-test comparing the two age groups directly. Table 1 summarizes the group differences in correlation patterns for each of the three seed points.

## Discussion

The present data indicate a crucial developmental difference in the default language network underlying sentence processing in childhood and adulthood. While adults display a network clearly lateralized in the left hemisphere, six-year-old children demonstrate stronger interhemispheric correlation to contralateral cortices. The adult default language network reveals fronto-temporal correlations within the left hemisphere both between left BA 44 and the left posterior STG/STS as well as between left IFS and the left posterior temporal cortex, with the latter correlation best distinguishing the adult group from the group of children. In adults, both the IFG and IFS are part of the network. Children's default language network, in contrast, is characterized by an absence of long-range functional connectivities between the inferior frontal cortex (IFG and IFS) and the posterior portions of the STS/STG, and the presence of stronger interhemispheric connectivities. This strong functional interhemispheric correlation might be based on the early structural maturation of the splenium as part of the corpus callosum connecting the two hemispheres [14], [15]. The splenium has been shown as the relevant part of the corpus callosum through which the auditory commissures project [39] and which is responsible for the interplay of left and right hemispheric language functions [40].

Our results indicate smaller correlations to distal VOIs in children compared to adults while at the same time local correlations around seed regions are larger. This observation is in line with previous rfMRI findings of more diffuse correlation patterns in children [41] and fMRI data of less specialized activation in pediatric compared to adult data [29]. Our data correspond to the view of a developing brain organization that assumes a shift in network architecture from a locally organized to a distally distributed pattern evolving during functional brain development [42], [43]. This observation was particularly found for seed 1 (BA 44, Figure 2) which may indicate a particularly late functional development in this specific area resulting in stronger effects in the group contrast.

The only fronto-temporal functional connectivity we observed in children was interhemispheric. This was a correlation from the left IFS to the right pSTG and supramarginal gyrus. This contralateral connection in children was potentially mediated via the precentral gyri bilaterally. We may speculate that the participation of the precentral gyri in the children's default language network reflects an involvement of the auditory-motor-mapping circuit hypothesized to be of particular functional relevance during language acquisition [6]. The observed contrahemispheric correlational pattern in children is in line with fMRI studies that report children's stronger reliance on the right hemisphere, reflected in a more rightward functional lateralization during language processing as compared to adults [29] and the age-related increase in the degree of lateralization [44], [45]. It has been discussed that the stronger involvement of the right hemisphere during language development may be due to a higher reliance on prosodic processes [46], [47] supported by the right hemisphere [48], [49]. Thus, the present LFF results indicate that the maturation of the default language network crucially depends on the increase of a functional connectivity between frontal and posterior temporal regions within the left hemisphere, and a decrease in contralateral interhemispheric connectivities.

The current neurophysiological findings, moreover, provide a link between existing behavioral data on the one hand, and structural connectivity data on the other. Behaviorally, children up to the age of seven are rather poor at comprehending syntactically complex sentences, although their comprehension of simple, subject-first sentences is close to perfect [50], [51], [52]. Structural connectivity data in children demonstrate that the dorsal pathway connecting the left frontal and posterior temporal cortex has not yet fully matured by seven years of age [17]. Together with the age-related differences observed in the functional connectivity, these findings indicate a certain immaturity of the language network before the age of seven, and suggest that the frontal-to-temporal connectivity between the language areas within the left hemisphere is a prerequisite for the processing of syntactically complex sentences.

The finding that the age-related difference in the frontal-to-posterior connectivity is most prominent for the functional connectivity between the IFS and temporal cortex, in addition to connectivity between BA 44 and the temporal cortex, points towards a crucial involvement of the IFS in the mature default language network. In adults, the left IFS has been shown to support aspects of working memory and to interact strongly with BA 44 during the processing of syntactically complex sentences [18]. In children, working memory capacities are taken as being crucial for success in dealing with complex syntax [53]. Therefore, the functional involvement of the IFS as part of a network processing complex sentences may be an important step towards the maturity of the default language network.

Thus, the development of the default language network from childhood to adulthood is characterised by a development from inter- to intrahemispheric connectivities with an increase in the long-range functional connectivities between the frontal and temporal regions within the left hemisphere. Given that a similar increase in the long-range connectivities is observed in the default network during resting state as the brain matures [26], [28], it is likely that this is a general principle underlying the normal development of cognitive processes [26], [54]. The current findings indicate that this principle clearly applies to the development of the default network for language.

## Materials and Methods

### Data Acquisition

For the present study, functional fMRI data from six-year-old children and adults using an established fMRI paradigm and material were analyzed [17], [29]. The data were collected at the Max Planck Institute for Human Cognitive and Brain Sciences, Leipzig, Germany, between 2006 and 2008. Participants received an MRI scan acquired on a 3T magnetic resonance scanner (Siemens Trio, Erlangen, Germany). Participants and caretakers gave written informed consent, children gave verbal consent. The study was approved by the Ethics Committee of the University of Leipzig.

The two experiments were part of a developmental study on language processing that investigated five- to seven-year-old children (N = 15, mean age 5.82, 5 to 7 yrs, 7 females) and adults (N = 16, mean age 26.2, 22 to 30 yrs, 8 females). Participants were right-handed German native speakers without neurological history and with normal language development. The paradigm used four conditions, two of which comprised correct sentences, one semantically incorrect sentences, and one syntactically incorrect sentences. These sentences were presented auditorily in a random fashion. Participants were asked to judge the acceptability of the sentences. Data from all four conditions entered the analysis. Data from both groups were acquired at the same scanner with identical protocols. Functional magnetic resonance images were acquired in a slab of 20 slices covering a central portion of the brain. A gradient-echo EPI sequence was used with TE 30 ms, flip angle 90 degrees, and TR = 2 seconds. The matrix acquired was 64×64 with an FOV of 19.2 cm, resulting in an in-plane resolution of 3×3 mm. The slice thickness was 4 mm with an inter-slice gap of 1 mm. Experiment length was 540 time steps for children and 900 time steps for adults. The datasets from the adults were cut after 540 for further data analysis. More detail on the experimental design and the data acquisition protocol are described in [29].

### Data Analysis

The analysis was done using the software package Lipsia [55] using the following processing steps. All data sets were initially corrected for motion by a 3D correction algorithm using 6 degrees of freedom (3 translational and 3 rotational). Data were aligned with the Talairach coordinate system while being resampled to a spatial resolution of (3 mm)^3^. In order to compensate for differences in brain size and shape between adults and children, all data sets were then non-linearly registered using the demon-matching algorithm [56] to a template brain which was selected based on the smallest amount of deviation from the group average of the overall sample. Following the removal of baseline drifts <0.0166 Hz, the data were further analyzed for correlations in the low-frequency domain between 0.1 Hz and 0.0166 Hz. No nuisance regressors or global covariates where applied [57], hence only positive correlations were observed and are reported.

In order to exclude effects due to stimulus onsets, we concentrated on the information contained in the residuals e of the general linear model [58] of the form KY = KXß+e, where Y denotes the measured time course in one voxel, X represents the design matrix that encoded the experimental stimulation convolved with a hemodynamic model based on the Gamma function, K is a Gaussian smoothing matrix with FWHM = 4 mm, and e is the residual error. We applied a low-pass filter to the residuals so that the subsequent analysis steps were restricted to low frequency fluctuations (LFFs) with frequencies below 0.0166 Hz.

Statistical examination concentrated on a VOI of perisylvian language areas of both hemispheres since a clear hypothesis for particular brain areas was formulated based on functional studies in adults covering different languages such as English, Italian, German and Hebrew as cited below. These areas were selected as a volume of interest. Analyses were conducted starting from three seed regions in areas that had been identified in functional fMRI studies as supporting sentence processing; the left dorsal IFG and the left posterior STG/STS. The IFG has frequently been reported to be engaged in syntactic processing [11], [18], [31], [32], [34], [35], [37], [59], as has the STG/STS [19], [33], [60], [61]. The coordinates for the seed regions were selected to cover left BA 44 (seed 1: −53, 20, 15), left IFS (seed 2: −47, 20, 30) and left STS (seed 3: −56, −43, 9), in order to best cover the regions involved in the processing of syntactically complex sentences. We defined the seed region as a sphere around the center points described above, comprising a volume of 7 voxels (189 mm^3^) each.

For each of these seed regions, we averaged the preprocessed fMRI time series across all 7 voxels and computed the Pearson's correlation coefficient of the averaged time course with all other voxels in the VOI mask for each data set. We normalized these correlations using Fisher's r-to-z transform z = 0.5 log((1+r)/(1−r)) to enforce Gaussianity of the correlation data and permit subsequent statistical tests. For each of the three seed regions, we performed voxelwise t-tests contrasting adult versus child data. Results were corrected for multiple comparisons using cluster-size and peak z-value thresholds obtained by Monte Carlo simulations [62]. Small-volume correction was applied within the VOI specified above. We hypothesized that the adult experiment would show a left-lateralized correlational pattern between the inferior frontal cortex and posterior temporal cortex when seeded in BA 44 and IFS, whereas the child experiment would only show weak frontal-to-temporal correlations. We also predicted strong left-lateralized correlations for adults between the posterior STS and the inferior frontal cortex when seeded in the STS, but a contralateral correlation in the temporal cortices in children. Since the current study covered the central portion of the brain, we cannot exclude that there is activation outside this region which warrants further investigation with whole brain fMRI.
